# Urinary Tissue Inhibitor of Metalloproteinase-2 (TIMP-2) • Insulin-Like Growth Factor-Binding Protein 7 (IGFBP7) Predicts Adverse Outcome in Pediatric Acute Kidney Injury

**DOI:** 10.1371/journal.pone.0143628

**Published:** 2015-11-25

**Authors:** Jens H. Westhoff, Burkhard Tönshoff, Sina Waldherr, Johannes Pöschl, Ulrike Teufel, Timm H. Westhoff, Alexander Fichtner

**Affiliations:** 1 Department of Pediatrics I, University Children’s Hospital, Heidelberg, Germany; 2 Department of Neonatology, University Children’s Hospital, Heidelberg, Germany; 3 Medizinische Klinik I, Universitätsklinik Marien Hospital Herne, Ruhr-University Bochum, Bochum, Germany; Centre Hospitalier Universitaire Vaudois, FRANCE

## Abstract

**Background:**

The G1 cell cycle inhibitors tissue inhibitor of metalloproteinase-2 (TIMP-2) and insulin-like growth factor-binding protein 7 (IGFBP7) have been identified as promising biomarkers for the prediction of adverse outcomes including renal replacement therapy (RRT) and mortality in critically ill adult patients who develop acute kidney injury (AKI). However, the prognostic value of urinary TIMP-2 and IGFBP7 in neonatal and pediatric AKI for adverse outcome has not been investigated yet.

**Methods:**

The product of the urinary concentration of TIMP-2 and IGFBP7 ([TIMP-2]•[IGFBP7]) was assessed by a commercially available immunoassay (NephroCheck^™^) in a prospective cohort study in 133 subjects aged 0–18 years including 46 patients with established AKI according to pRIFLE criteria, 27 patients without AKI (non-AKI group I) and 60 apparently healthy neonates and children (non-AKI group II). AKI etiologies were: dehydration/hypovolemia (n = 7), hemodynamic instability (n = 7), perinatal asphyxia (n = 9), septic shock (n = 7), typical hemolytic-uremic syndrome (HUS; n = 5), interstitial nephritis (n = 5), vasculitis (n = 4), nephrotoxic injury (n = 1) and renal vein thrombosis (n = 1).

**Results:**

When AKI patients were classified into pRIFLE criteria, 6/46 (13%) patients fulfilled the criteria for the category “Risk”, 13/46 (28%) for “Injury”, 26/46 (57%) for “Failure” and 1/46 (2%) for “Loss”. Patients in the “Failure” stage had a median 3.7-fold higher urinary [TIMP-2]•[IGFBP7] compared to non-AKI subjects (P<0.001). When analyzed for AKI etiology, highest [TIMP-2]•[IGFBP7] values were found in patients with septic shock (P<0.001 vs. non-AKI I+II). Receiver operating characteristic (ROC) curve analyses in the AKI group revealed good performance of [TIMP-2]•[IGFBP7] in predicting 30-day (area under the curve (AUC) 0.79; 95% CI, 0.61–0.97) and 3-month mortality (AUC 0.84; 95% CI, 0.67–0.99) and moderate performance in predicting RRT (AUC 0.67; 95% CI, 0.50–0.84).

**Conclusions:**

This study shows that urinary [TIMP-2]•[IGFBP7] has a good diagnostic performance in predicting adverse outcomes in neonatal and pediatric AKI of heterogeneous etiology.

## Introduction

Acute kidney injury (AKI) is a complex disorder that constitutes an independent risk factor for morbidity and mortality in adult and pediatric patients [1, 2]. Approximately 10% of all children admitted to a pediatric intensive care unit (PICU) develop AKI, and this rate exceeds 80% with increasing severity of illness [3, 4]. In critically ill children, the reported mortality from AKI is still as high as 60% [5]. The frequency and spectrum of AKI etiologies differ between pediatric and adult patients. Of note, pediatric AKI epidemiological data revealed a shift from primary renal diseases to renal involvement secondary to other systemic diseases, particularly in hospitalized children [6, 7]. Furthermore, AKI survivors appear to be at significant short- and long-term risk for complications such as chronic kidney disease (CKD) [8].

Changes in serum creatinine (SCr) and/or urine output form the basis of diagnostic and staging criteria for AKI [9]. However, SCr is a late and unspecific marker of reduced glomerular filtration rate and is insensitive to acute changes in kidney function [10]. Neither the cause and location of the renal disease (e.g. prerenal versus intrinsic; affected renal tubule segment; nephrotoxic versus ischemic AKI) nor the extent of renal damage are adequately reflected by SCr concentrations [9]. Eventually, SCr is influenced by several non-renal factors such as muscle mass, medications, diet and tubular secretion, thus provoking inaccuracies in AKI diagnosis. Hence, current research aims at providing better diagnostic tools for detection, specification and prognosis of AKI. This includes (i) early diagnosis of kidney damage before renal function has severely diminished, (ii) differentiation of functional (prerenal) versus intrinsic AKI, (iii) identification of AKI patients at risk for severe and long-lasting kidney damage and for other adverse outcomes [11, 12]. Over the last decade, significant progress has been made in the identification and validation of novel biomarkers for AKI [12]. Several of them also proved valuable for use in pediatric AKI [13]. Recent studies reported on the discovery and validation of two urinary G1 cell cycle arrest biomarkers for early diagnosis of kidney damage: tissue inhibitor of metalloproteinase-2 (TIMP-2) and insulin-like growth factor-binding protein 7 (IGFBP7) [14–16]. Following injury, renal tubular cells enter a period of G1 cell-cycle arrest that is assumed to protect cells from dividing once DNA damage has occurred [17, 18]. In consequence, this will lead to either cellular repair including reconstitution of genomic integrity, cell death or cellular senescence [19–22]. Urinary [TIMP-2]•[IGFBP7] performed better than any other biomarker reported to date for predicting the development of moderate or severe AKI according to Kidney Disease Improving Global Outcomes (KDIGO) criteria in patients at high risk for AKI from multiple causes [23]. Furthermore, the risk for death, dialysis or persistent renal dysfunction in AKI patients increased with higher [TIMP-2]•[IGFBP7] values [16]. Recently, a secondary analysis from the Sapphire study in adults demonstrated that [TIMP-2]•[IGFBP7] determined early in the setting of critical illness identified AKI patients at increased risk for mortality or requirement for renal replacement therapy (RRT) within 9 months following AKI [24].

The performance of urinary [TIMP-2]•[IGFBP7] in neonatal and pediatric AKI for the prediction of adverse outcomes has never been tested. This prompted us to investigate the value of urinary [TIMP-2]•[IGFBP7] in an etiologically heterogenous cohort of patients with established AKI according to pRIFLE criteria for the prediction of adverse clinical outcomes.

## Material and Methods

### Ethics Statement

The study was approved by the local ethics committee of the Medical Faculty of Heidelberg. Written informed consent was obtained by legal guardian of each patient with assent from the patient when appropriate. The clinical investigation was conducted according to the principles expressed in the Declaration of Helsinki.

### Study Design and Participants

We performed a prospective cohort study at the University Children’s Hospital Heidelberg. Patients aged 0 to 18 years referred to our clinic with established AKI and patients who developed AKI during hospitalization were consecutively enrolled in the study from October 2011 to September 2014. AKI was classified according to the pediatric-modified Risk, Injury, Failure, Loss, and Endstage renal disease (pRIFLE) AKI criteria [3]. Exclusion criteria were prematurity, hereditary nephropathies and postrenal AKI as examined by initial renal ultrasound. Etiological assignments of AKI were established by three independent physicians. Noteworthy, two AKI patients of our study cohort suffered from CKD stage 2 and presented with prerenal AKI (“acute on chronic”) due to hypovolemia. One patient was kidney-transplanted due to congenital renal hypoplasia and dysplasia, suffered from chronic allograft nephropathy and also presented with prerenal AKI due to hypovolemia. One patient was excluded from our study cohort, as he was stem cell transplanted and received a graft versus host disease (GVHD) prophylaxis that included cyclosporine which has been shown previously to increase the urinary TIMP-2/creatinine ratio [25].

Control subjects without AKI were taken from two different cohorts: the first group (non-AKI group I, total: n = 27) consisted of non-AKI patients either admitted to our neonatal or pediatric intensive-care unit (NICU/PICU) (n = 17) or who presented for out-patient treatment with minor medical conditions such as constipation (n = 10). Diagnoses of NICU/PICU non-AKI patients were: postoperative care (n = 15), seizures (n = 1), pneumonia (n = 1). In order to identify a potential impact of non-renal illnesses on urinary [TIMP-2]•[IGFBP7] in non-AKI group I, we investigated another control group consisting of apparently healthy children aged 0–18 years (non-AKI group II, n = 60). Exclusion criteria were: (a) Any known or suspected acute illness at the time of enrollment; (b) any known or suspected chronic disease; (c) any surgery within the last 6 months; (d) any blood product transfusion within the previous 2 months.

### Sample and data collection

Urine samples were obtained immediately after admission to our hospital or—in case of anuria—after restoration of diuresis. Urine samples were centrifuged and the supernatant was frozen, stored in aliquots at −80°C and thawed prior to analysis. SCr, C-reactive protein (CrP) and urinalysis were measured as part of the routine clinical chemistry testing. Measurement of SCr was performed using an IDMS-traceable enzymatic method. The estimated creatinine clearance (eCCl) for children was calculated by using the revised Schwartz formula: (k = 0.413 × height/serum creatinine) [26]. For determination of baseline eCCl, the lowest value of SCr in the 3 months prior to study enrollment was obtained from the hospital database. In case of unknown baseline serum creatinine and absence of previous kidney injuries in the case history, the patient was assumed to have normal renal function and assigned a baseline eCCl of 120 ml/min per 1.73 m^2^ [27]. In addition, for term neonates who developed AKI within the first days of life, i.e. before SCr reached baseline levels, an eCCl of 40 ml/min per 1.73 m^2^ was assigned [28–30]. eCCl was used for pRIFLE_CCl_ classification with “Risk” defined as eCCl decrease of 25% from baseline, “Injury” defined as eCCl decrease of 50%, “Failure” defined as eCCl decrease of 75% or absolute value <35 ml/min per 1.73 m2 and “Loss” as persistent renal failure > 4 weeks. Of note, neonates were diagnosed as “Failure” only if eCCl decreased by at least 75% and not based on an absolute eCCl value <35 ml/min per 1.73 m^2^. In addition, pRIFLE_uo_ stage was determined by urinary output criteria [3]. For this, urine output was systematically and continuously evaluated every 2 hours during the initial period by age-appropriate techniques. As newborns can be oliguric during the first 24 hours of life, a reduction in urinary output was not considered a criterion in this time period. The maximum score was taken for pRIFLE stage assignment.

### Laboratory analysis

The respective concentrations of TIMP-2 and IGFBP7 were measured in spot urine samples using the NephroCheck^TM^ Test (Astute Medical, San Diego, CA, USA). Urine TIMP-2 and IGFBP7 are simultaneously measured with this point-of-care test. [TIMP-2]•[IGFBP7] indicates the product (by multiplication) of the respective urinary concentration of both biomarkers that is automatically calculated by the ASTUTE140^®^ Meter. The product is divided by 1,000 to report a single numerical test result with a unit of (ng/ml)^2^/1000, the unit for all [TIMP-2]•[IGFBP7] values in this report.

### Statistical analysis

Distribution of numerical data was analyzed by the Kolmogorov-Smirnov Test. Not normally distributed data are presented as median and interquartile range. Comparison of not normally distributed numerical data was performed by the Mann—Whitney U-test or the Kruskal-Wallis-test with post-hoc Dunn's Test, respectively (age, SCr on study enrollment, eCCl on study enrollment, proteinuria, urine protein-to-creatinine ratio, CrP, length of ICU stay, length of hospital stay, [TIMP-2]•[IGFBP7] for AKI and non-AKI groups, [TIMP-2]•[IGFBP7] for age-groups, [TIMP-2]•[IGFBP7] for pRIFLE stages, [TIMP-2]•[IGFBP7] for AKI etiologies, [TIMP-2]•[IGFBP7] for comparison of asphyctic newborns with non-AKI newborns). Comparison of categorical parameters was performed by Fisher’s exact test in case of dichotomy and by Pearson chi-squared test in case of polychotomy (gender, proportion of neonates per group, RRT, 30 day-mortality, 3 month-mortality). P < 0.05 was regarded as statistically significant. To analyze the predictive power of [TIMP-2]•[IGFBP7], receiver operating characteristic (ROC) curves were calculated and the area under the ROC curve (AUC) was determined. 95% confidence intervals (CI) were reported. The [TIMP-2]•[IGFBP7] value maximizing the Youden index was used to derive sensitivity, specificity, positive predictive value (PPV) and negative predictive value (NPV), assuming sensitivity and specificity were equally important. The composite outcome of death or dialysis within 3 months was assessed using Kaplan—Meier estimates and log-rank test for comparison between the groups. Descriptive statistics, statistical tests, ROC curve and Kaplan-Meier analyses were performed using IBM^®^ SPSS^®^ Statistics Version 22.

## Results

### Subject characteristics

One-hundred thirty-three subjects were enrolled in the study including 46 AKI patients, 27 patients without AKI (non-AKI group I) and 60 apparently healthy control children (non-AKI group II). The epidemiological data, etiologies of AKI, pRIFLE stages and renal laboratory parameters are presented in Table 1. There was no statistically significant difference regarding age, gender distribution and proportion of neonates between the three groups. The distribution of AKI causes reflects the typical spectrum of a PICU in a tertiary care University Children’s Hospital (Table 1). The median duration from the onset of AKI to study enrollment was 2.0 (1.0 to 5.0) days. SCr on study enrollment was significantly higher in the AKI group compared to the non-AKI group I and eCCl on study enrollment was markedly decreased in the AKI group compared to the non-AKI group I (Table 1). In addition, the AKI group displayed significantly higher values for CrP, urinary protein concentration and urinary protein-to-creatinine ratio compared to the non-AKI group I (P < 0.001). The length of the ICU stay was 4-fold and the length of hospitalitation 1.7-fold higher in the AKI group compared to the non-AKI group I (Table 1). Sixteen of 46 AKI patients (34.8%) received RRT, 14 (87.5%) hemodialysis and 2 (12.5%) peritoneal dialysis. Seven patients (15.2%) deceased within 3 months following study enrollment, six of them (13.0%) within the first 30 days. Causes of death were septic shock with coexisting AKI (n = 5), cerebral incarceration in perinatal asphyxia (n = 1) and low-cardiac output (n = 1). On day of demise two of the patients received RRT, and all suffered from AKI according to pRIFLE criteria. At the time of study enrollment, 6 patients (13.0%) fullfilled the “Risk”, 13 patients (28.3%) the “Injury”, 26 (56.5%) the “Failure” and 1 patient (2.2%) the “Loss” criteria. No patient developed end-stage renal disease. During the clinical course, 4 patients progressed to the next higher pRIFLE stage.

**Table 1:** Characteristics of the study population.

	Total (n = 133)	AKI group (n = 46)	Non-AKI group I (n = 27)	Non-AKI group II (n = 60)	*P-value*
**Age (years)**	3.4 (0.0 to 9.0)	1.2 (0.0 to 10.5)	2.4 (0.6 to 6.5)	5.5 (0.0 to 9.0)	0.79
**Male**	58 (43.6%)	17 (37.0%)	11 (40.7%)	30 (50.0%)	0.38
**Female**	75 (56.4%)	29 (63.0%)	16 (59.3%)	30 (50.0%)	
**Neonates**	36 (27.1%)	14 (30.4%)	4 (14.8%)	18 (30.0%)	0.28
**AKI etiology**					
Hypovolemia/dehydration		7 (15.2%)			
Hemodynamic instability		7 (15.2%)			
Perinatal asphyxia		9 (19.6%)			
Septic shock		7 (15.2%)			
Typical HUS		5 (10.9%)			
Interstitial nephritis		5 (10.9%)			
Vasculitis		4 (8.7%)			
Nephrotoxic insult		1 (2.2%)			
Renal vein thrombosis		1 (2.2%)			
**SCr on study enrollment (mg/dL)**		1.7 (0.9 to 3.4)	0.3 (0.2 to 0.5)		**< 0.001**
**SCr at discharge from hospital (mg/dL)**		0.4 (0.3 to 0.7)			
**eCCl on study enrollment (mL/min per 1.73 m** ^**2**^ **)**		18.9 (10.5 to 29.2)	128.5 (99.7 to 154.0)		**< 0.001**
**pRIFLE stage on study enrollment**					
Risk		6 (13.0%)			
Injury		13 (28.3%)			
Failure		26 (56.5%)			
Loss		1 (2.2%)			
**Maximum pRIFLE stage**					
Risk		5 (10.9%)			
Injury		11 (23.9%)			
Failure		29 (63.0%)			
Loss		1 (2.2%)			
**Proteinuria (g/L)**		0.4 (0.1 to 1.7)	0.1 (0.0 to 0.1)		**< 0.001**
**Urinary protein-to-creatinine ratio (g/mol creatinine)**		212.4 (69.0 to 602.1)	26.7 (9.8 to 43.8)		**< 0.001**
**CrP (mg/L)**		28.8 (4.2 to 94.3)	0.0 (0.0 to 2.4)		**< 0.001**
**RRT**		16 (34.8%)	0 (0%)	0 (0%)	**< 0.001**
Hemodialysis		14 (87.5%)			
Peritoneal dialysis		2 (12.5%)			
**30-day mortality**		6 (13.0%)	0 (0%)	0 (0%)	**0.003**
**3-month mortality**		7 (15.2%)	0 (0%)	0 (0%)	**0.001**
**Time period from onset of AKI to study enrollment (days)**		2.0 (1.0 to 5.0)			
**Length of ICU stay (days)**		10.0 (6.5 to 21.0)	2.5 (1.0 to 13.8)		**0.009**
**Length of hospitalization (days)**		16.0 (10.3 to 34.0)	9.5 (4.5 to 17.0)		**0.007**
**Urinary [TIMP-2]•[IGFBP7]**	0.28 (0.10 to 0.77)	0.69 (0.22 to 3.27)	0.10 (0.06 to 0.41)	0.27 (0.10 to 0.49)	**< 0.001**

Numeric data are presented as median and interquartile range due to non-normal distribution. Statistical tests used for the individual parameters are presented in the statistics section. Unit for [TIMP-2]•[IGFBP7] is (ng/mL)²/1,000. Abbreviations: AKI, acute kidney injury; SCr, serum creatinine; eCCl, estimated creatinine clearance; CrP, C-reactive protein; RRT, renal replacement therapy; ICU, intensive care unit.

Subanalyses of the subject characteristics were performed for the group of neonates (S1 Table) and the group of children > 28 days of life (S2 Table). In addition, SCr values on study enrollment were analyzed for different age groups (0–28 days, 29 days—2 years, 2–5 years, 6–11 years, 12–18 years) in AKI and non-AKI subjects and are listed in S3 Table.

As shown in S4 Table, perinatal asphyxia (66.7%) was prevalent in patients at the “Risk” stage. In the “Injury” stage, the most frequent AKI etiologies were hemodynamic instability (38.5%), hypovolemia/dehydration (30.8%) and perinatal asphyxia (23.1%), and in the “Failure” stage septic shock (26.9%), interstitial nephritis (19.2%), typical HUS (15.4%), hypovolemia/dehydration (11.5%) and vasculitis (11.5%).

### Urinary [TIMP-2]•[IGFBP7] stratified for age in subjects with or without AKI

**Fig 1:**
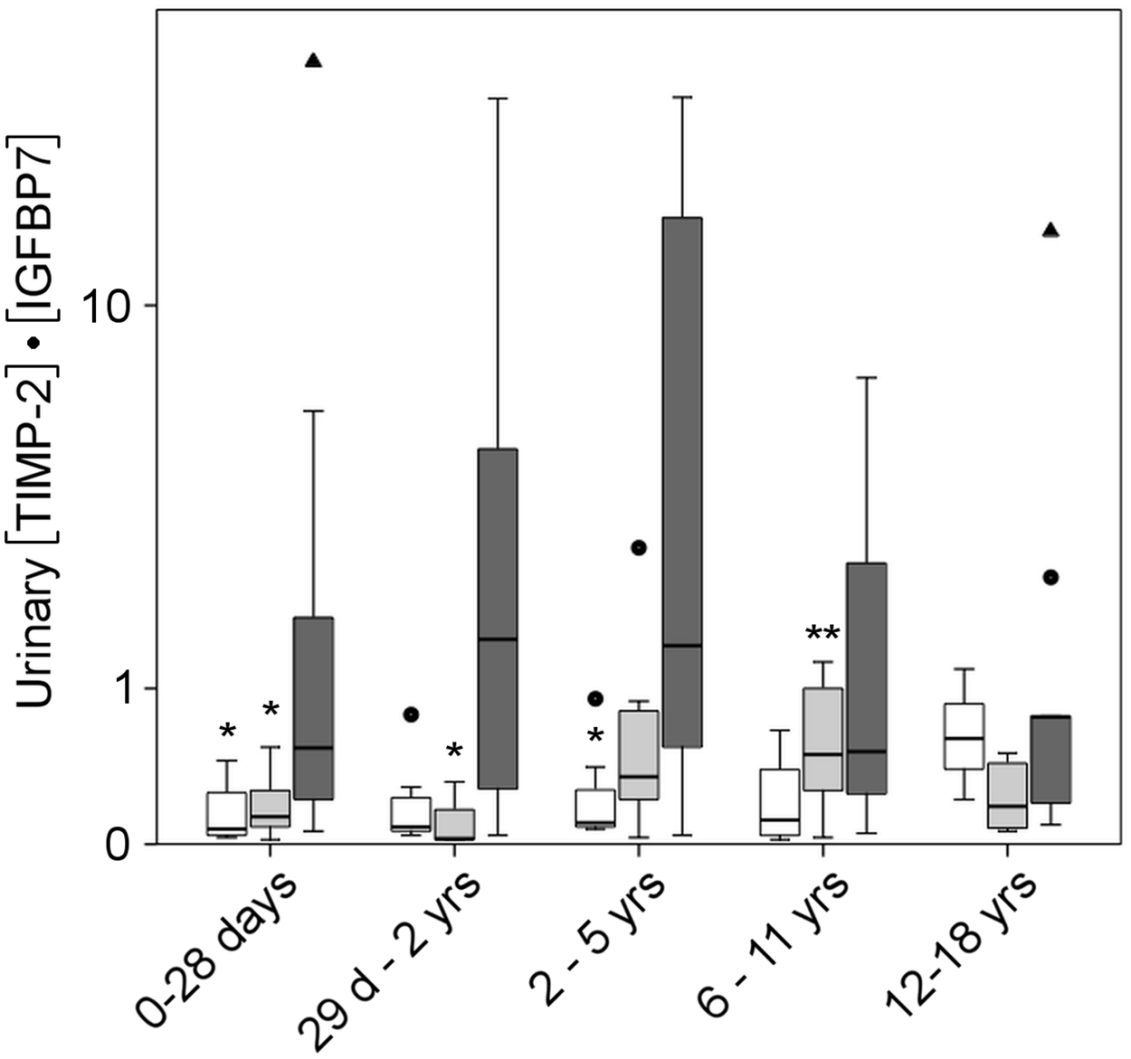
Urinary [TIMP-2]•[IGFBP7] in non-AKI and AKI subjects stratified for age. Boxplots of urinary [TIMP-2]•[IGFBP7] for different age ranges for the non-AKI group I (white boxes), non-AKI group II (light grey boxes) and the AKI group (dark grey boxes). Selected age groups were composed as follows: 0–28 days (non-AKI group I, n = 4; non-AKI group II, n = 18; AKI group, n = 14), 29 days—2 years (non-AKI group I, n = 9; non-AKI group II, n = 4; AKI group, n = 10), 2–5 years (non-AKI group I, n = 7; non-AKI group II, n = 13; AKI group, n = 9), 6–11 years (non-AKI group I, n = 4; non-AKI group II, n = 17; AKI group, n = 4), 12–18 years (non-AKI group I, n = 3; non-AKI group II, n = 8; AKI group, n = 9). The lower and upper edges of the box represent the first and third quartile, respectively, while the horizontal line within the box indicates the median. The vertical length of the box represents the interquartile range (IQR). The most extreme sample values (within a distance of 1.5 x IQR) are the endpoints of the whiskers. Outliers (1.5–3.0 x IQR outside the box) are shown as dots, extremes (> 3.0 x IQR) as triangles. Unit for [TIMP-2]•[IGFBP7] is (ng/mL)^2^/1,000. Abbreviations: d, days; yrs, years. *P<0.05 vs. AKI of same age group, **P<0.05 vs. age group 0–28 days and 29 days– 2 years of non-AKI group II by Kruskal-Wallis test and Dunn’s multiple comparison test.

Median urinary [TIMP-2]•[IGFBP7] was numerically, but not significantly (P = 0.41) higher in the non-AKI group II compared to the non-AKI group I (Table 1) and significantly increased in the AKI group (P<0.001 vs. non-AKI group I; P = 0.001 vs. non-AKI-group II). As shown in Fig 1 and S5 Table, urinary [TIMP-2]•[IGFBP7] values were subsequently analyzed for the following age groups: 0–28 days, 29 days—2 years, 2–5 years, 6–11 years and 12–18 years. Urinary [TIMP-2]•[IGFBP7] was not significantly different among these five age groups in both non-AKI cohorts; however, there seemed to be a tendency towards lower values in younger children and neonates. If appropriate, [TIMP-2]•[IGFBP7] data of non-AKI group I and II patients were combined for further analyses (non-AKI group I+II).

As shown in Fig 1 and S5 Table, urinary [TIMP-2]•[IGFBP7] values were significantly increased in the AKI group compared to the non-AKI groups for the following age groups: 0–28 days, 29 days—2 years and 2–5 years.

### Urinary [TIMP-2]•[IGFBP7] stratified for pRIFLE and for AKI etiologies

**Fig 2:**
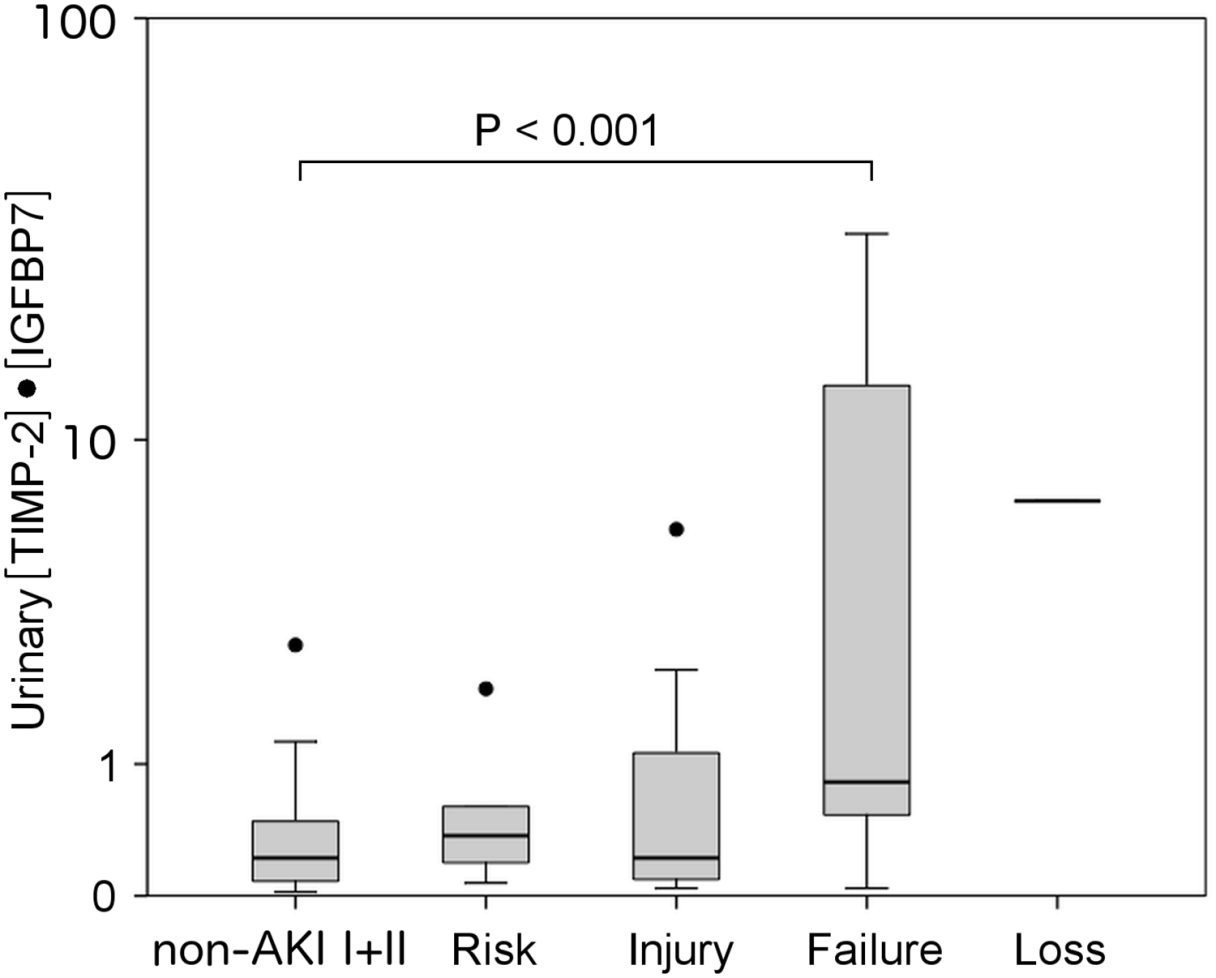
Urinary [TIMP-2]•[IGFBP7] in established AKI according to pRIFLE classification. Boxplots of urinary [TIMP-2]•[IGFBP7] for the AKI cohort (n = 46) stratified for the different pRIFLE stages. Groups were composed as follows: non-AKI I+II (n = 87), “Risk” (n = 6), “Injury” (n = 13), “Failure” (n = 26), “Loss” (n = 1). For explanation of boxplots and unit for [TIMP-2]•[IGFBP7] see legend of Fig 1. P<0.001 for “Failure” vs. non-AKI I+II by Kruskal-Wallis test and Dunn’s multiple comparison test.

**Fig 3:**
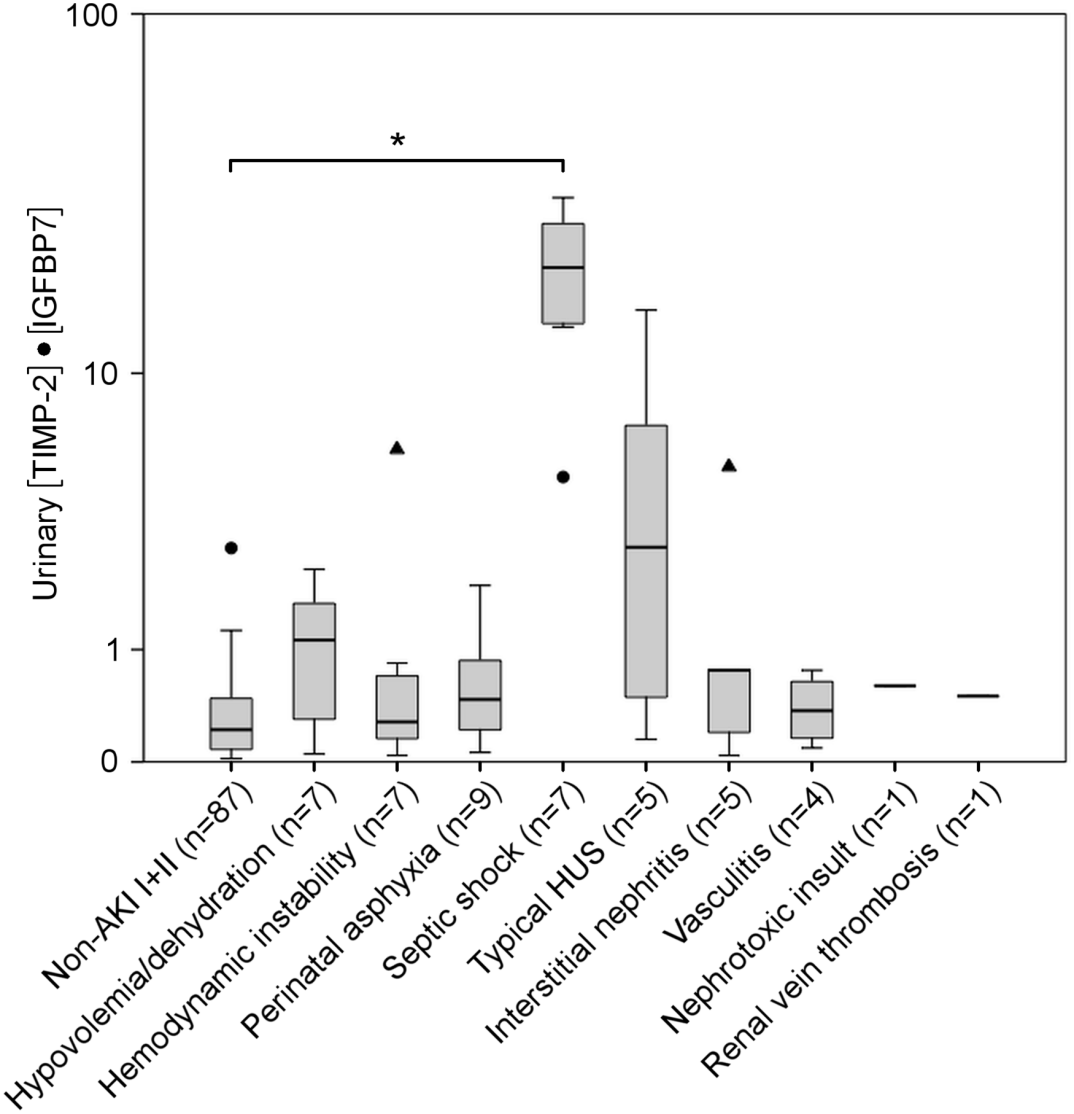
Urinary [TIMP-2]•[IGFBP7] in established AKI of heterogeneous etiology. Boxplots of urinary [TIMP-2]•[IGFBP7] for the different AKI etiologies. Cause of AKI is listed under the boxplot, number of patients of each group in brackets. For explanation of boxplots and unit for [TIMP-2]•[IGFBP7] see legend of Fig 1. Abbreviations: HUS, hemolytic uremic syndrome. *P<0.001 by Kruskal-Wallis test and Dunn’s multiple comparison test.

When stratified for pRIFLE criteria, there was a significant increase in urinary [TIMP-2]•[IGFBP7] for the “Failure” stage (P < 0.001) compared to non-AKI group I+II (Fig 2 and S4 Table). The increase in urinary [TIMP-2]•[IGFBP7] remained statistically significant for the “Failure” stage when neonates and older children were analyzed separately (S6 Table). When [TIMP-2]•[IGFBP7] was analyzed for the underlying AKI etiology (Fig 3, S7 Table), AKI in septic shock patients was associated with the highest [TIMP-2]•[IGFBP7] values (P<0.001) compared to non-AKI group I+II. Subsequently, non-significant increases in [TIMP-2]•[IGFBP7] were found for typical HUS (P = 0.51), hypovolemia/dehydration, interstitial nephritis, nephrotoxic AKI, renal vein thrombosis, perinatal asphyxia and vasculitis. Asphyctic newborns with AKI had 4.3-fold (P = 0.02) higher urinary [TIMP-2]•[IGFBP7] values (0.47, 0.15 to 1.31; n = 9) than newborns of non-AKI group I+II (0.11, 0.06 to 0.27; n = 22). ROC curve analysis of [TIMP-2]•[IGFBP7] for the assessment of AKI severity revealed an AUC of 0.75 (95% CI: 0.65–0.85) for the pRIFLE stage “Injury” and higher and an AUC of 0.74 (95% CI: 0.64–0.83) for any pRIFLE stage.

### Prognostic Accuracy of urinary [TIMP-2]•[IGFBP7] in predicting mortality and RRT

**Fig 4:**
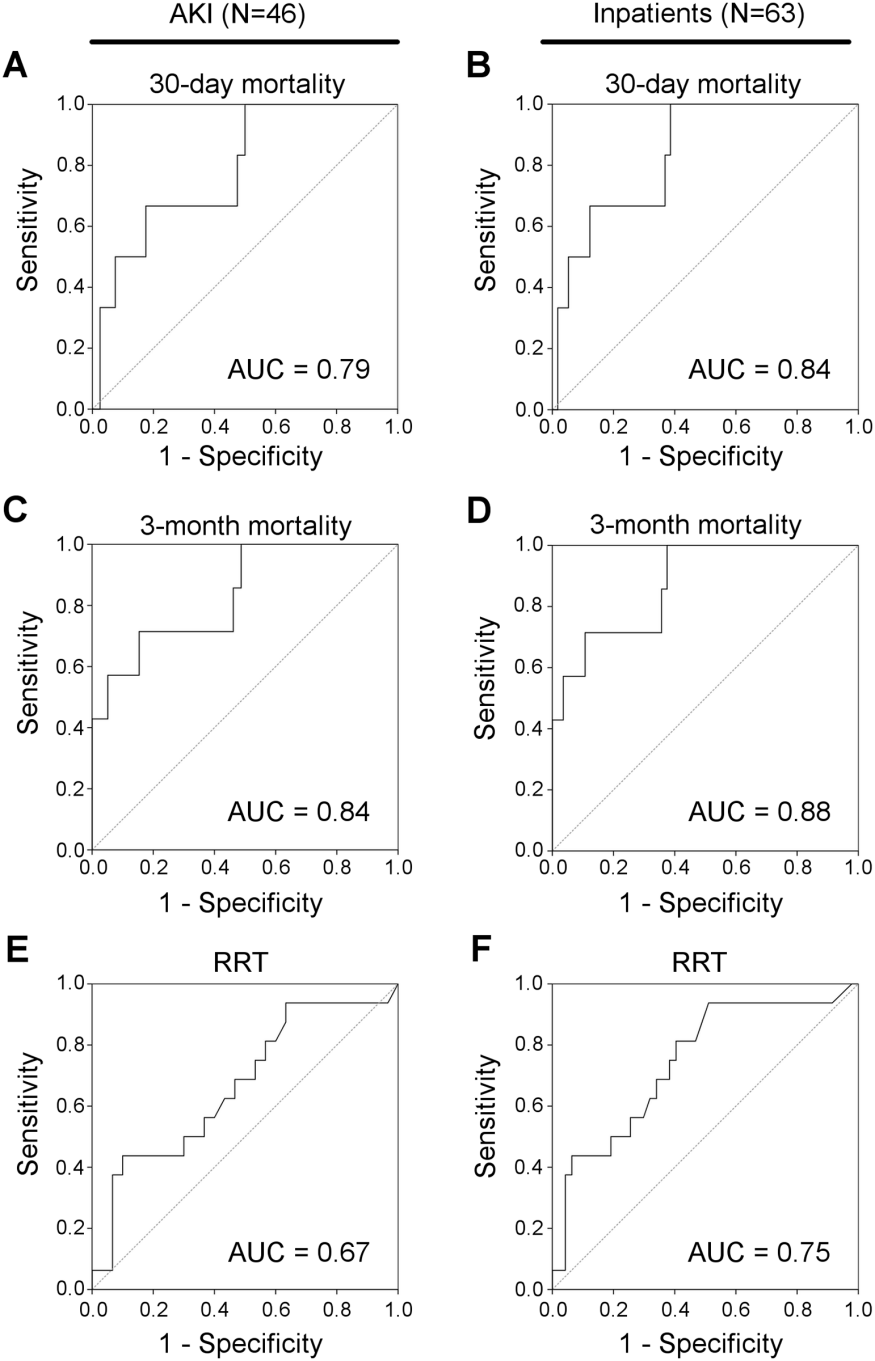
Diagnostic accuracy of [TIMP-2]•[IGFBP7] for the prediction of adverse outcomes in AKI. Receiver operating characteristic curves for the diagnostic accuracy of [TIMP-2]•[IGFBP7] in prediction of 30-day mortality, 3-month mortality and need for RRT assessed for the AKI group (A, C, E) and the group of inpatients (AKI patients plus ICU patients without AKI; N = 63) (B, D, F).

As shown in Fig 4, ROC curve analysis for the accuracy of [TIMP-2]•[IGFBP7] in prediction of 30-day mortality revealed an AUC of 0.79 (95% CI: 0.61–0.97) when analyzed for the AKI group and an AUC of 0.84 (95% CI: 0.70–0.98) for all inpatients, i.e. AKI patients and non-AKI PICU/NICU patients (n = 63). Regarding the prediction of 3-month mortality ROC curve analysis revealed an AUC of 0.84 (95% CI: 0.67–1.00) for the AKI group and an AUC of 0.88 (95% CI: 0.75–1.00) for all inpatients. Optimal [TIMP-2]•[IGFBP7] cut-off values were calculated for prediction of 30-day mortality within the AKI group according to the Youden index. For a threshold [TIMP-2]•[IGFBP7] value of 0.56, sensitivity was 100% and specificity was 50%. The PPV and NPV were 23.1% and 100%, respectively. Regarding 3-month mortality, for a threshold [TIMP-2]•[IGFBP7] value of 3.78, sensitivity was 71.4% and specificity was 84.6%. PPV and NPV were 45.5% and 94.3%, respectively.

When the AKI group was analyzed for [TIMP-2]•[IGFBP7] in predicting RRT, the AUC was 0.67 (95% CI: 0.50–0.84) (Fig 4). When all inpatients (n = 63) were analyzed, the AUC was 0.75 (95% CI: 0.60–0.89). ROC curve analysis was subsequently used for the AKI group to determine the optimal cut-off value according to the Youden index for the differentiation of non-RRT and RRT patients. [TIMP-2]•[IGFBP7] achieved a sensitivity of 43.8% and a specificity of 93.6% for a threshold value of 4.99. The resulting PPV and NPV were 70.0% and 83.0%, respectively.

In addition, prognostic accuracy of [TIMP-2]•[IGFBP7] for prediction of mortality and RRT was separately analyzed for neonates and children (S8 Table). In neonates, ROC curve analyses revealed lower accuracies of [TIMP-2]•[IGFBP7] for prediction of 30-day and 3-month mortality compared to the combined neonatal and pediatric group, whereas prognostic accuracy for prediction of RRT was increased. Of note, only one patient of this group received dialysis (S1 Table). The group of older children demonstrated excellent accuracies of [TIMP-2]•[IGFBP7] for prediction of 30-day and 3-month, however accuracy for prediction of RRT was only poor to fair.

To reduce the risk of a sampling bias in the neonatal population by application of the pRIFLE classification that was originally developed in children above the age of one month [3], this age-group was additionally stratified for the neonatal modified KDIGO AKI definition [31]. As shown in S9 Table, the ROC curve analysis for the accuracy of [TIMP-2]•[IGFBP7] in predicting 30-day and 3-month mortality and the requirement for RRT revealed comparable results to those obtained by use of the pRIFLE classification. Of note, only 8 neonates were assigned as having AKI by the neonatal modified KDIGO classification in comparison to 14 neonatal patients as defined by the pRIFLE classification.

### Increased risk of death or dialysis at increased [TIMP-2]•[IGFBP7] cut-off values in pediatric AKI patients

**Fig 5:**
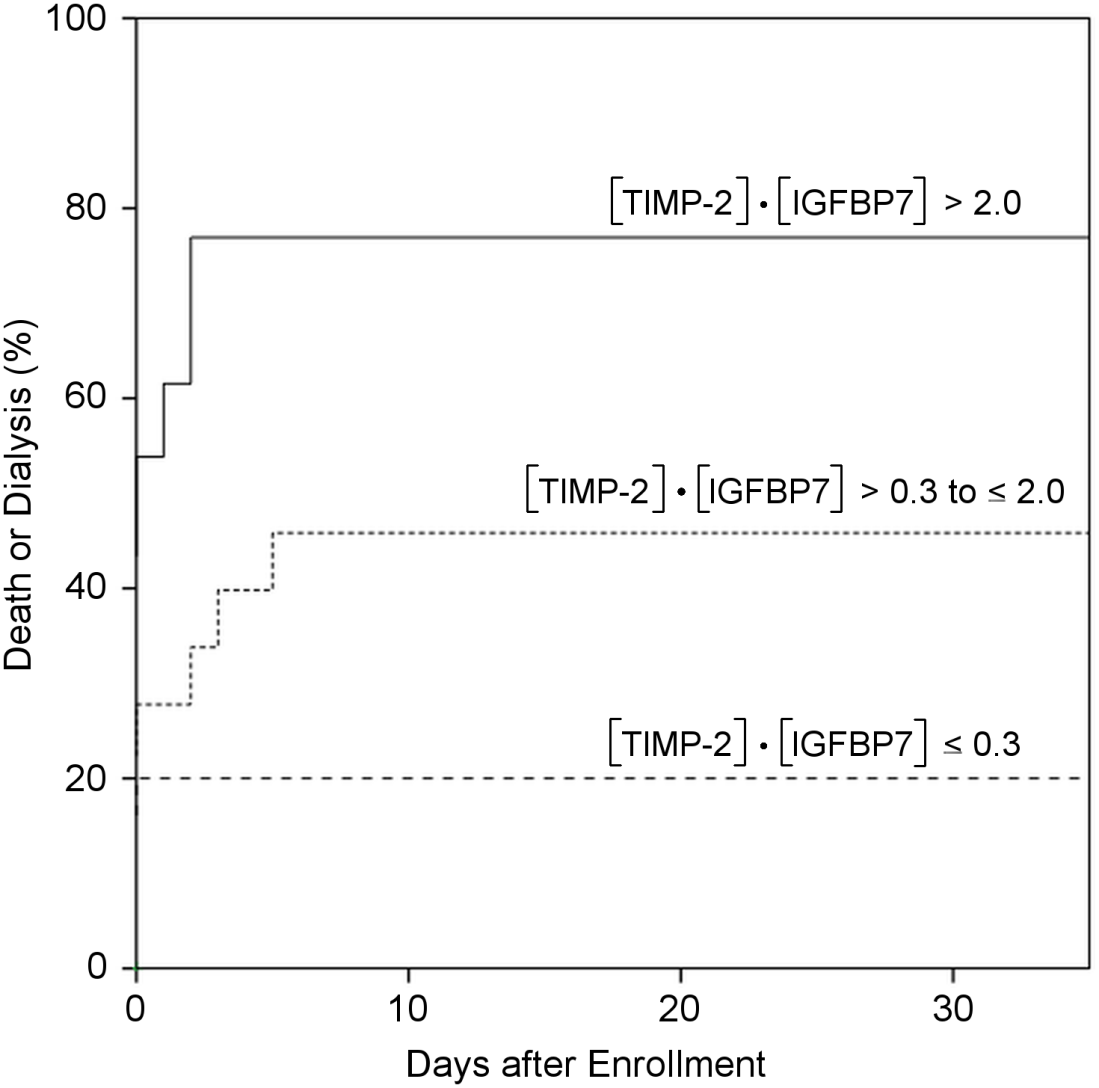
Kaplan—Meier curves for death or dialysis within 3 months after study enrollment. Composite endpoint of death or dialysis within 3 months for patients with established AKI (pRIFLE “Risk” stage or higher). A time period of only 30 days is shown as all first events of the composite endpoint occurred within this time period. Urinary [TIMP-2]**•**[IGFBP7] ranges of ≤0.3 (long dashed line, n = 15), 0.3 to ≤2.0 (short dashed line, n = 18), and >2.0 (solid line, n = 13) are shown. Log-rank P = 0.003 for [TIMP-2]**•**[IGFBP7] >2.0 vs. [TIMP-2]**•**[IGFBP7] ≤0.3.

AKI patients were stratified for selected [TIMP-2]•[IGFBP7] cut-off values that were previously published for adults [14]. Kaplan-Meier analyses were performed for the composite end point of death or dialysis within 3 months (Fig 5). AKI patients with urinary [TIMP-2]•[IGFBP7] values > 2.0 revealed a significantly increased risk for death or dialysis compared to AKI patients with [TIMP-2]•[IGFBP7] values ≤ 0.3 (P = 0.003). In a multivariable cox proportional hazards model, [TIMP-2]•[IGFBP7] values > 2.0 were independently associated with a significantly increased risk of RRT or death (HR 2.91; 95% CI: 1.19–7.08; P = 0.019). As decision for initiation of RRT can be subjective, Kaplan-Meier curves were also performed for mortality within 3 months as the end point. [TIMP-2]•[IGFBP7] values > 2.0 remained significantly associated with an increased risk compared to [TIMP-2]•[IGFBP7] values ≤ 0.3 (P = 0.009; data not shown).

## Discussion

This is the first study investigating urinary [TIMP-2]•[IGFBP7] in neonatal and pediatric primary and secondary AKI of heterogeneous etiology other than the likewise homogeneous setting of cardiac surgery with cardiopulmonary bypass [32]. Our findings show that once-only measurement of [TIMP-2]•[IGFBP7] in patients with AKI has a good prognostic performance in predicting adverse outcomes such as mortality and a moderate performance in predicting the requirement for RRT.

While in recent years a major focus has been the identification of novel biomarkers for prediction of incipient AKI, less attention was paid to the search for informative biomarkers predictive of AKI outcome, including renal recovery, need for RRT and patient death [33]. In a proteomic approach in a small cohort of critically ill adult patients, IGFBP7 and neutrophil gelatinase-associated lipocalin (NGAL) were identified as best predictors of renal recovery; subsequent validation confirmed the prognostic value of IGFBP7 and NGAL in predicting mortality (IGFBP7: AUC 0.68; NGAL: AUC 0.81), recovery (IGFBP7: AUC 0.74; NGAL: AUC 0.70) and severity of AKI (IGFBP7: AUC 0.77; NGAL: AUC 0.69) [34]. Kashani et al. demonstrated that the risk for major adverse kidney events including death, RRT or persistent renal dysfunction within 30 days increased considerably for [TIMP-2]•[IGFBP7] above 0.3 and doubled when values were > 2.0 [16]. Interestingly, a recently published secondary analysis of the Sapphire study revealed that a single measurement of [TIMP-2]•[IGFBP7] early in the setting of critical illness is able to identify adult patients with AKI at increased risk for mortality or receipt of RRT over the subsequent 9 months [24]. Our data demonstrate that an once-only urinary [TIMP-2]•[IGFBP7] measurement is a good predictor for 30 day- (AUC 0.79) and 3 month-mortality (AUC 0.84) and a fair predictor for RRT (AUC 0.67) in a heterogeneous neonatal and pediatric cohort of established primary and secondary AKI according to pRIFLE criteria. The lower performance of urinary [TIMP-2]•[IGFBP7] for the prediction of RRT may be due to the fact that the indication for RRT is physician-dependent and not coherently defined. In addition, by Kaplan-Meier and cox proportional hazard model analyses for the composite end point “death or dialysis” within 3 months we show that patients with AKI and [TIMP-2]•[IGFBP7] values > 2.0 are at higher risk for adverse outcomes than AKI patients with [TIMP-2]•[IGFBP7] values ≤ 0.3. This result is in accordance with recently published data by Koyner et al. [24] who demonstrated an increased risk for adverse outcomes with increasing [TIMP-2]•[IGFBP7]. Of note, in that study (Sapphire) urine samples were obtained before AKI, as defined by KDIGO AKI criteria, was established. In summary, our findings support the usefulness of once-only [TIMP-2]•[IGFBP7] measurements for predicting adverse outcomes in AKI in neonatal and pediatric patients.

Age-related reference values for neonates and children are indispensable to interprete presumably pathological results, yet have not been evaluated for urinary [TIMP-2]•[IGFBP7]. We therefore analyzed [TIMP-2]•[IGFBP7] in two different control groups, namely one group consisting of patients without AKI and another group consisting of apparently healthy neonates and children. Reference values were largely stable among the different age groups and comparable to adults [16], but tended to be lower in neonates and younger children. There was no statistically significant difference between both control groups, arguing against an effect of extrarenal disease or other hospital-associated conditions on [TIMP-2]•[IGFBP7] values in the non-AKI patient group (non-AKI group I). Hence, in addition to adults [16], our data from neonates and children emphasizes that urinary [TIMP-2]•[IGFBP7] appears to be specific for AKI, in contrast to several other biomarkers [16]. For some age groups (0–28 days, 2–5 years, 6–11 years) apparently healthy neonates and children showed moderately higher urinary [TIMP-2]•[IGFBP7] values compared to the non-AKI patients. As urine samples were in most cases obtained a few hours before centrifugation and transported to our clinic by the parents, pre-analytical sample handling might be a source of error. Further research is required to explain the variability of urinary [TIMP-2]•[IGFBP7] values. Meersch et al. showed that pediatric patients with congenital heart disease displayed relatively high baseline (before cardiac surgery) urinary [TIMP-2]•[IGFBP7] values (mean 0.9–1.0). Twenty-four hours post-surgery, values dropped to 0.4–0.5, which indicates that either preoperative venous congestion or fasting have an effect on renal integrity [32].

When urinary [TIMP-2]•[IGFBP7] was categorized for the different pRIFLE stages and compared to non-AKI patients and apparently healthy controls, increased [TIMP-2]•[IGFBP7] values were significantly associated with the “Failure” stage (P<0.001). Lower patient numbers in the “Risk” and “Injury” group and a higher percentage of short-term renal injuries (e.g. hypovolemia/dehydration and hemodynamic instability) are potential explanations for the only moderate increase of [TIMP-2]•[IGFBP7] for the “Risk” and “Injury” stage. When analyzed for the different AKI etiologies, the strongest increase in [TIMP-2]•[IGFBP7] was found in septic shock. All septic shock patients with AKI fulfilled “Failure” criteria according to the pRIFLE classification. Both intrarenal hemodynamic changes as well as nonhemodynamic mediators of injury (e.g. inflammatory cytokines, oxidative stress, arachidonic acid metabolites, vasoactive substances) contribute to renal damage in sepsis and septic shock [35]. Thus, the multitude of cascades involved in tubular damage might explain markedly elevated [TIMP-2]•[IGFBP7] values when compared to other AKI causes. That septic AKI is rather a protracted than a short-term process could be another explanation for high [TIMP-2]•[IGFBP7] values in this subgroup. Increases in urinary [TIMP-2]•[IGFBP7] for the other AKI etiologies showed no statistical significance. Further research with higher patient numbers is required to unravel the role of [TIMP-2]•[IGFBP7] in different AKI subtypes.

There are several limitations of our study. First, this is a single-center study in a relatively small patient cohort, which requires validation in a larger patient population. Second, our study cohort includes both neonates and children. Due to varying degrees of creatinine reabsorption in the proximal tubules, overall lower but rapidly increasing GFRs, inter-individual maturational differences and interference with maternal creatinine values within the first days of life, a SCr/eCCl-based definition of AKI in neonates has to be regarded with caution. This is especially the case when using the pRIFLE classification and eCCl estimates for neonates with missing baseline SCr values. Eventually, an estimated baseline eCCl of either 40 ml/min per 1.73 m^2^ for neonates or 120 ml/min per 1.73 m^2^ for children aged > 1 month might lead to misclassification. In addition, the pRIFLE classification was originally developed in children > 1 month of age [3]. Therefore, we performed subanalyses for the group of neonates and the group of children, which independently confirmed the main findings of our study. Of note, the accuracy of [TIMP-2]•[IGFBP7] in predicting 30-day and 3-month mortality further increased after exclusion of the neonates. To minimize the risk of a sampling bias in the neonatal group owing to AKI assignment based on the pRIFLE criteria, this group was additionally classified using the increasingly accepted neonatal modified KDIGO AKI definition [31]. In spite of a reduced number of newborns classified as AKI, the prognostic accuracy for the prediction of adverse outcomes remained largely unchanged (S9 Table). Considering the characteristics of neonatal renal function and the difficulties regarding clinical diagnosis and prognosis using current laboratory values (i.e. SCr), the search for new biomarkers of kidney injury is of particular interest for this age group. A third limitation of our study is the heterogeneity of the patient population, which comprised different subtypes of AKI. Fourthly, as several patients were transferred to our clinic with already established AKI, the time interval between onset of AKI and study enrollment was variable. As the respective half-life of these biomarkers is short, we might have missed earlier increases in [TIMP-2]•[IGFBP7].

## Conclusions

Our data show that once-only measurement of [TIMP-2]•[IGFBP7] has a good diagnostic performance in predicting mortality and a moderate performance in predicting requirement for RRT in a combined cohort of neonatal and pediatric AKI. Urinary [TIMP-2]•[IGFBP7] values are largely independent of age in non-AKI patients and apparently healthy neonates and children. Increased [TIMP-2]•[IGFBP7] values are associated with the extent of renal damage as reflected by the pRIFLE classification.

## Supporting Information

S1 Table Characteristics of the neonatal study population. (DOCX)

S2 Table Characteristics of the pediatric study population > 28 days of age. (DOCX)

S3 Table SCr on study enrollment stratified for age in AKI and non-AKI patients. (DOCX)

S4 Table Urinary [TIMP-2]•[IGFBP7] classified by pRIFLE stages. (DOCX)

S5 Table Age-related urinary [TIMP-2]•[IGFBP7] values in AKI and non-AKI patients. (DOCX)

S6 Table Urinary [TIMP-2]•[IGFBP7] in neonates and children stratified for pRIFLE stage. (DOCX)

S7 Table Urinary [TIMP-2]•[IGFBP7] in neonates and children classified for different AKI etiologies. (DOCX)

S8 Table Diagnostic accuracy of [TIMP-2]•[IGFBP7] for the prediction of adverse outcomes in neonates and children. (DOCX)

S9 Table Diagnostic accuracy of urinary [TIMP-2]•[IGFBP7] for the prediction of adverse outcomes in the neonatal AKI group stratified for either pRIFLE or neonatal modified KDIGO AKI definition. (DOCX)
