# Supplementary material for: Urinary Tissue Inhibitor of Metalloproteinase-2 (TIMP-2) • Insulin-Like Growth Factor-Binding Protein 7 (IGFBP7) Predicts Adverse Outcome in Pediatric Acute Kidney Injury
Source: PLoS One. 2015 Nov 25;10(11):e0143628. doi: 10.1371/journal.pone.0143628 (PMC4659607; doi:10.1371/journal.pone.0143628)
Supplement: S1 Table — (DOCX) [file pone.0143628.s001.docx]

**S1 Table.** Characteristics of the neonatal study population.

|  | **AKI group**  **(n=14)** | **Non-AKI group I (n=4)** | **Non-AKI group II (n=18)** | ***P-value*** |
| --- | --- | --- | --- | --- |
| **Age (days)** | 2.0 (1.0 to 4.3) | 3.5 (2.3 to 10.0) | 1.0 (1.0 to 2.0) | **0.006** |
| **Male**  **Female** | 7 (50 %)  7 (50 %) | 1 (25 %)  3 (75 %) | 11 (61 %)  7 (39 %) | 0.410 |
| **AKI etiology:** |  |  |  |  |
| **Hypovolemia/dehydration**  **Hemodynamic instability**  **Perinatal asphyxia**  **Septic shock**  **Typical HUS**  **Interstitial nephritis**  **Vasculitis**  **Nephrotoxic insult**  **Renal vein thrombosis** | 0 (0 %)  3 (21.4 %)  9 (64.3 %)  1 (7.1 %)  0 (0 %)  0 (0 %)  0 (0 %)  0 (0 %)  1 (7.1 %) |  |  |  |
| **SCr on study enrollment (mg/dL)** | 1.2 (0.8 to 2.4) | 0.3 (0.2 to 0.5) |  | **< 0.001** |
| **SCr at discharge from hospital (mg/dL)** | 0.4 (0.3 to 0.6) |  |  |  |
| **eCCl on study enrollment (mL/min per 1.73 m^2^)** | 17.6 (9.5 to 25.3) | 71.2 (46.7 to 125.5) |  | **< 0.001** |
| **pRIFLE stage on study enrollment** | R: n=5 (35.7 %)  I: n=4 (28.6 %)  F: n=5 (35.7 %) |  |  |  |
| **Maximum pRIFLE stage** | R: n=5 (35.7 %)  I: n=4 (28.6 %)  F: n=5 (35.7 %) |  |  |  |
| **Proteinuria (mg/L)** | 0.17 (0.13 to 0.99) | 0.08 (0.05 to 0.10) |  | **< 0.001** |
| **Urinary protein-to-creatinine ratio (mg/g)** | 375.1 (109.9 to 699.8) | 58.1 (39.9 to 63.1) |  | **< 0.001** |
| **CrP (mg/L)** | 11.9 (5.9 to 39.7) | 3.5 (0.6 to 12.8) |  | **< 0.001** |
| **RRT** | 1/14 (34.0%)  HD: n=1 | 0/4 (0%) | 0/18 (0%) |  |
| **30 day-mortality** | 2/14 (14.3 %) | 0/4 (0%) | 0/18 (0%) | 0.189 |
| **3 month-mortality** | 3/14 (21.4 %) | 0/4 (0%) | 0/18 (0%) | 0.076 |
| **Time period from onset of AKI to study enrollment (days)** | 1.0 (0.8 to 3.0) |  |  |  |
| **Length of ICU stay (days)** | 8.0 (6.5 to 11.5) | 13.0 (5.0 to 22.0) |  | **0.009** |
| **Length of hospitalization (days)** | 16.0 (11.5 to 21.0) | 11.5 (7.0 to 26.5) |  | **0.007** |
| **Urinary [TIMP-2]•[IGFBP7]** | 0.54 (0.21 to 1.80) | 0.07 (0.04 to 0.36) | 0.13 (0.08 to 0.27) | **0.004** |

Numeric data are presented as median and interquartile range due to non-normal distribution. Statistical tests used for the individual parameters are presented in the statistics section. Unit for [TIMP-2]•[IGFBP7] is (ng/mL)²/1,000. Abbreviations: AKI, acute kidney injury; R, Risk; I, Injury; F, Failure; SCr, serum creatinine; eCCl, estimated creatinine clearance; CrP, C-reactive protein; RRT, renal replacement therapy; HD, hemodialysis; ICU, intensive care unit.
