# Supplementary material for: Urinary Tissue Inhibitor of Metalloproteinase-2 (TIMP-2) • Insulin-Like Growth Factor-Binding Protein 7 (IGFBP7) Predicts Adverse Outcome in Pediatric Acute Kidney Injury
Source: PLoS One. 2015 Nov 25;10(11):e0143628. doi: 10.1371/journal.pone.0143628 (PMC4659607; doi:10.1371/journal.pone.0143628)
Supplement: S2 Table — (DOCX) [file pone.0143628.s002.docx]

**S2 Table.** Characteristics of the pediatric study population > 28 days of age.

|  | **AKI group**  **(n=32)** | **Non-AKI group I (n=23)** | **Non-AKI group II (n=42)** | ***P-value*** |
| --- | --- | --- | --- | --- |
| **Age (years)** | 5.3 (0.9 to 13.1) | 3.4 (0.9 to 7.0) | 8.0 (4.8 to 11.3) | **0.017** |
| **Male**  **Female** | 10 (31.3 %)  22 (68.8 %) | 10 (43.5 %)  13 (56.5 %) | 19 (45.2 %)  23 (54.8 %) | 0.447 |
| **AKI etiology:** |  |  |  |  |
| **Hypovolemia/dehydration**  **Hemodynamic instability**  **Perinatal asphyxia**  **Septic shock**  **Typical HUS**  **Interstitial nephritis**  **Vasculitis**  **Nephrotoxic insult**  **Renal vein thrombosis** | 7 (21.9 %)  4 (12.5 %)  0 (0 %)  6 (18.8 %)  5 (15.6 %)  5 (15.6 %)  4 (12.5 %)  1 (3.1 %)  0 (0 %) |  |  |  |
| **SCr on study enrollment (mg/dL)** | 2.4 (1.1 to 4.4) | 0.3 (0.2 to 0.5) |  | **< 0.001** |
| **SCr at discharge from hospital (mg/dL)** | 0.5 (0.2 to 0.8) |  |  |  |
| **eCCl on study enrollment (mL/min per 1.73 m^2^)** | 19.2 (11.0 to 36.5) | 130.9 (101.9 to 156.6) |  | **< 0.001** |
| **pRIFLE stage on study enrollment** | R: n=1 (3.1 %)  I: n=9 (28.1 %)  F: n=21 (65.6 %)  L: n=1 (3.1 %) |  |  |  |
| **Maximum pRIFLE stage** | I: n=7 (21.9 %)  F: n=24 (75.0 %)  L: n=1 (3.1 %) |  |  |  |
| **Proteinuria (mg/L)** | 0.48 (0.11 to 2.01) | 0.05 (0.03 to 0.08) |  | **< 0.001** |
| **Urinary protein-to-creatinine ratio (mg/g)** | 173.2 (45.9 to 637.3) | 18.5 (9.5 to 40.0) |  | **< 0.001** |
| **CrP (mg/L)** | 33.5 (1.1 to 98.8) | 0.0 (0.0 to 0.0) |  | **< 0.001** |
| **RRT** | 15/32 (46.9 %)  HD: n=13 (86.7 %)  PD: n=2 (13.3 %) | 0/23 (0%) | 0/42 (0%) |  |
| **30 day-mortality** | 4/32 (12.5 %) | 0/23 (0%) | 0/42 (0%) | **0.014** |
| **3 month-mortality** | 4/32 (12.5 %) | 0/23 (0%) | 0/42 (0%) | **0.014** |
| **Time period from onset of AKI to study enrollment (days)** | 3.0 (1.0 to 7.0) |  |  |  |
| **Length of ICU stay (days)** | 15.5 (6.5 to 37.0) | 1.0 (1.0 to 10.0) |  | **0.003** |
| **Length of hospitalization (days)** | 16.0 (10.0 to 39.0) | 8.0 (4.0 to 16.0) |  | **0.007** |
| **Urinary [TIMP-2]•[IGFBP7]** | 0.77 (0.21 to 5.08) | 0.14 (0.06 to 0.41) | 0.34 (0.17 to 0.57) | **< 0.001** |

Numeric data are presented as median and interquartile range due to non-normal distribution. Statistical tests used for the individual parameters are presented in the statistics section. Unit for [TIMP-2]•[IGFBP7] is (ng/mL)²/1,000. Abbreviations: AKI, acute kidney injury; R, Risk; I, Injury; F, Failure; L, Loss; SCr, serum creatinine; eCCl, estimated creatinine clearance; CrP, C-reactive protein; RRT, renal replacement therapy; HD, hemodialysis; PD, peritoneal dialysis; ICU, intensive care unit.
