# Supplementary material for: Urinary Tissue Inhibitor of Metalloproteinase-2 (TIMP-2) • Insulin-Like Growth Factor-Binding Protein 7 (IGFBP7) Predicts Adverse Outcome in Pediatric Acute Kidney Injury
Source: PLoS One. 2015 Nov 25;10(11):e0143628. doi: 10.1371/journal.pone.0143628 (PMC4659607; doi:10.1371/journal.pone.0143628)
Supplement: S3 Table — (DOCX) [file pone.0143628.s003.docx]

**S3 Table.** SCr on study enrollment stratified for age in AKI and non-AKI patients.

|  | **AKI group (n=46)** | **Non-AKI group I (n=27)** | ***P-value*** |
| --- | --- | --- | --- |
| 0 – 28 days (n=18) | 1.20 (0.80 to 2.36) [n=14] | 0.31 (0.19 to 0.49) [n=4] | **0.001** |
| 29 days – 2 years (n=19) | 1.08 (0.73 to 1.83) [n=10] | 0.22 (0.20 to 0.30) [n=9] | **< 0.001** |
| 2-5 years (n=16) | 1.57 (0.74 to 3.19) [n=9] | 0.27 (0.22 to 0.31] [n=7] | **< 0.001** |
| 6-11 years (n=8) | 5.61 (2.59 to 12.52) [n=4] | 0.44 (0.35 to 0.47) [n=4] | **0.029** |
| 12-18 years (n=12) | 4.58 (2.83 to 7.60) [n=9] | 0.60 (0.53 to 0.78) [n=3] | **0.009** |

Numeric data are presented as median and interquartile range due to non-normal distribution. Median and upper and lower value are shown for the group of n=3. Unit for SCr is mg/dL. Abbreviations: AKI, acute kidney injury. Statistical analysis was performed by Mann-Whitney-U-Test.
