# Supplementary material for: Urinary Tissue Inhibitor of Metalloproteinase-2 (TIMP-2) • Insulin-Like Growth Factor-Binding Protein 7 (IGFBP7) Predicts Adverse Outcome in Pediatric Acute Kidney Injury
Source: PLoS One. 2015 Nov 25;10(11):e0143628. doi: 10.1371/journal.pone.0143628 (PMC4659607; doi:10.1371/journal.pone.0143628)
Supplement: S4 Table — (DOCX) [file pone.0143628.s004.docx]

**S4 Table.** Urinary [TIMP-2]•[IGFBP7] classified by pRIFLE stages.

|  | **Non-AKI group I+II**  **(n=87)** | **Risk (n=6)** | **Injury (n=13)** | **Failure (n=26)** | **Loss (n=1)** |
| --- | --- | --- | --- | --- | --- |
| **AKI etiology** |  | Perinatal asphyxia:  n=4 (66.7%)  Hemodynamic instability:  n=1 (16.7%)  Nephrotoxic insult:  n=1 (16.7%) | Hemodynamic instability:  n=5 (38.5%)  Hypovolemia/  dehydration:  n=4 (30.8%)  Perinatal asphyxia:  n=3 (23.1%)  Vasculitis:  n=1 (7.7%) | Septic shock:  n=7 (26.9%)  Interstitial nephritis  n=5 (19.2%)  Typical HUS:  n=4 (15.4%)  Hypovolemia/  dehydration:  n=3 (11.5%)  Vasculitis:  n=3 (11.5%)  Perinatal asphyxia:  n=2 (7.7%)  Hemodynamic instability:  n=1 (3.8%)  Renal vein thrombosis:  n=1 (3.8%) | Typical HUS:  n=1 (100%) |
| **Urinary**  **[TIMP-2]•[IGFBP7]** | 0.22 (0.08-0.48) | 0.38 (0.16-0.94) | 0.22 (0.08-1.43) | 0.82 (0.52-13.79)* | 6.97 |

Numeric data are presented as median and interquartile range due to non-normal distribution. The unit for [TIMP-2]•[IGFBP7] is (ng/mL)²/1,000. Abbreviations: AKI, acute kidney injury; HUS, hemolytic uremic syndrome. *P<0.001 for “Failure” vs. non-AKI I+II by Kruskal-Wallis test and Dunn’s multiple comparison test.
