# Supplementary material for: Urinary Tissue Inhibitor of Metalloproteinase-2 (TIMP-2) • Insulin-Like Growth Factor-Binding Protein 7 (IGFBP7) Predicts Adverse Outcome in Pediatric Acute Kidney Injury
Source: PLoS One. 2015 Nov 25;10(11):e0143628. doi: 10.1371/journal.pone.0143628 (PMC4659607; doi:10.1371/journal.pone.0143628)
Supplement: S5 Table — (DOCX) [file pone.0143628.s005.docx]

**S5 Table.** Age-related urinary [TIMP-2]•[IGFBP7] values in AKI and non-AKI patients.

|  | **Non-AKI group I (n=27)** | **Non-AKI group II (n=60)** | **AKI group (n=46)** | ***P-value*** |
| --- | --- | --- | --- | --- |
| **0–28 days (n=36)** | 0.07 (0.04 to 0.36)* [n=4] | 0.13 (0.08 to 0.27)* [n=18] | 0.54 (0.21 to 1.80) [n=14] | **0.004** |
| **29 days - 2 years (n=23)** | 0.08 (0.05 to 0.26) [n=9] | 0.03 (0.02 to 0.25)* [n=4] | 1.52 (0.24 to 7.01) [n=10] | **0.007** |
| **2-5 years (n=29)** | 0.10 (0.07 to 0.41)* [n=7] | 0.35 (0.20 to 0.84) [n=13] | 1.42 (0.32 to 17.68) [n=9] | **0.046** |
| **6-11 years (n=25)** | 0.12 (0.03 to 0.54) [n=4] | 0.49 (0.26 to 1.07)** [n=17] | 0.51 (0.16 to 5.36) [n=4] | 0.264 |
| **12-18 years (n=20)** | 0.60 (0.22 to 1.18) [n=3] | 0.19 (0.07 to 0.46) [n=8] | 0.76 (0.18 to 1.53) [n=9] | 0.112 |

Numeric data are presented as median and interquartile range due to non-normal distribution. Median and upper and lower value are shown for the group of n=3. Unit for [TIMP-2]•[IGFBP7] is (ng/mL)²/1,000. *P<0.05 vs. AKI subjects of same age group. **P<0.05 vs. 0-28 days and P<0.05 vs. 29 days – 2 years of non-AKI group II. Abbreviations: AKI, acute kidney injury. Statistical analysis was performed by Kruskal-Wallis test and Dunn’s multiple comparison test.
