# Supplementary material for: Urinary Tissue Inhibitor of Metalloproteinase-2 (TIMP-2) • Insulin-Like Growth Factor-Binding Protein 7 (IGFBP7) Predicts Adverse Outcome in Pediatric Acute Kidney Injury
Source: PLoS One. 2015 Nov 25;10(11):e0143628. doi: 10.1371/journal.pone.0143628 (PMC4659607; doi:10.1371/journal.pone.0143628)
Supplement: S6 Table — (DOCX) [file pone.0143628.s006.docx]

**S6 Table.** Urinary [TIMP-2]•[IGFBP7] in neonates and children stratified for pRIFLE stage.

|  | **Neonates (n=36)** | **Children (n=97)** |
| --- | --- | --- |
| **Non-AKI group I+II** | 0.11 (0.06 to 0.27) [n=22] | 0.25 (0.08 to 0.55) [n=65] |
| **Risk** | 0.28 (0.13 to 1.22) [n=5] | 0.60 [n=1] |
| **Injury** | 0.98 (0.10 to 4.84) [n=4] | 0.12 (0.07 to 0.98) [n=9] |
| **Failure** | 0.62 (0.54 to 16.19) [n=5]* | 1.42 (0.51 to 13.95) [n=21]** |
| **Loss** |  | 6.97 [n=1] |

Numeric data are presented as median and interquartile range due to non-normal distribution. Unit for [TIMP-2]•[IGFBP7] is (ng/mL)²/1,000. Abbreviations: AKI, acute kidney injury. *P<0.01 vs. neonatal non-AKI group I+II. **P<0.01 vs. pediatric non-AKI group I+II. Statistical analysis was performed by Kruskal-Wallis test and Dunn’s multiple comparison test.
