# Supplementary material for: Urinary Tissue Inhibitor of Metalloproteinase-2 (TIMP-2) • Insulin-Like Growth Factor-Binding Protein 7 (IGFBP7) Predicts Adverse Outcome in Pediatric Acute Kidney Injury
Source: PLoS One. 2015 Nov 25;10(11):e0143628. doi: 10.1371/journal.pone.0143628 (PMC4659607; doi:10.1371/journal.pone.0143628)
Supplement: S7 Table — (DOCX) [file pone.0143628.s007.docx]

**S7 Table.** Urinary [TIMP-2]•[IGFBP7] in neonates and children classified for different AKI etiologies.

| **AKI etiology** | **[TIMP-2]•[IGFBP7]** | ***P-value***  *(vs. non-AKI*  *group I+II)* |
| --- | --- | --- |
| Hypovolemia/dehydration (n=7) | 1.12 (0.10 to 1.92) | 1.000 |
| Hemodynamic instability (n=7) | 0.28 (0.12 to 0.84) | 1.000 |
| Perinatal asphyxia (n=9) | 0.47 (0.15 to 1.31) | 1.000 |
| Septic shock (n=7) | 20.12 (13.63 to 26.76) | **< 0.001** |
| Typical HUS (n=5) | 2.76 (0.32 to 11.11) | 0.513 |
| Interstitial nephritis (n=5) | 0.76 (0.12 to 2.97) | 1.000 |
| Vasculitis (n=4) | 0.38 (0.13 to 0.70) | 1.000 |
| Nephrotoxic insult (n=1) | 0.60 | 1.000 |
| Renal vein thrombosis (n=1) | 0.50 | 1.000 |
| Non-AKI group I+II (n=87) | 0.22 (0.08 to 0.48) |  |

Data are presented as median and interquartile range due to non-normal distribution. The unit for [TIMP-2]•[IGFBP7] is (ng/mL)²/1,000. Abbreviations: AKI, acute kidney injury; HUS, hemolytic uremic syndrome. Statistical analysis was performed by Kruskal-Wallis test and Dunn’s multiple comparison test.
