# Supplementary material for: Urinary Tissue Inhibitor of Metalloproteinase-2 (TIMP-2) • Insulin-Like Growth Factor-Binding Protein 7 (IGFBP7) Predicts Adverse Outcome in Pediatric Acute Kidney Injury
Source: PLoS One. 2015 Nov 25;10(11):e0143628. doi: 10.1371/journal.pone.0143628 (PMC4659607; doi:10.1371/journal.pone.0143628)
Supplement: S8 Table — (DOCX) [file pone.0143628.s008.docx]

**S8 Table.** Diagnostic accuracy of [TIMP-2]•[IGFBP7] for the prediction of adverse outcomes in neonates and children.

|  | **AKI (n=46)** | | **Inpatients (n=63)** | |
| --- | --- | --- | --- | --- |
|  | **Neonates (n=14)** | **Children (n=32)** | **Neonates (n=18)** | **Children (n=45)** |
| **30-day mortality** | 0.58 (95% CI: 0.30-0.86) | 0.94 (95% CI: 0.84-1.00) | 0.69 (95% CI: 0.46-0.92) | 0.96 (95% CI: 0.89-1.00) |
| **3-month mortality** | 0.76 (95% CI: 0.48-1.00) | 0.94 (95% CI: 0.84-1.00) | 0.82 (95% CI: 0.61-1.00) | 0.96 (95% CI: 0.89-1.00) |
| **RRT** | 1.00 (95% CI: 1.00-1.00) | 0.61 (95% CI: 0.41-0.81) | 1.00 (95% CI: 1.00-1.00) | 0.72 (95% CI: 0.56-0.88) |

Data are presented as area under the curve (AUC) value and 95% confidence interval (CI) obtained from receiver operating characteristic (ROC) curve analysis. Abbreviations: AKI, acute kidney injury; ROC, receiver operating characteristic; RRT, renal replacement therapy.
