# Supplementary material for: Urinary Tissue Inhibitor of Metalloproteinase-2 (TIMP-2) • Insulin-Like Growth Factor-Binding Protein 7 (IGFBP7) Predicts Adverse Outcome in Pediatric Acute Kidney Injury
Source: PLoS One. 2015 Nov 25;10(11):e0143628. doi: 10.1371/journal.pone.0143628 (PMC4659607; doi:10.1371/journal.pone.0143628)
Supplement: S9 Table — (DOCX) [file pone.0143628.s009.docx]

**S9 Table.** Diagnostic accuracy of urinary [TIMP-2]•[IGFBP7] for the prediction of adverse outcomes in the neonatal AKI group stratified for either pRIFLE or neonatal modified KDIGO AKI definition.

|  | **AKI** | | |  | **Inpatients** | | |
| --- | --- | --- | --- | --- | --- | --- | --- |
|  | 30-day mortality | 3-month mortality | RRT |  | 30-day mortality | 3-month mortality | RRT |
| pRIFLE  [Akcan-Arikan et al., 2007] | 0.58 (95% CI: 0.30-0.86) [n=14] | 0.76 (95% CI: 0.48-1.00) [n=14] | 1.00 (95% CI: 1.00-1.00) [n=14] |  | 0.69 (95% CI: 0.46-0.92) [n=18] | 0.82 (95% CI: 0.61-1.00) [n=18] | 1.00 (95% CI: 1.00-1.00) [n=18] |
| Neonatal modified KDIGO  [Selewski et al., 2015] | 0.50 (95% CI: 0.10-0.90) [n=8] | 0.73 (95% CI: 0.36-1.00) [n=8] | 1.00 (95% CI: 1.00-1.00) [n=8] |  | 0.70 (95% CI: 0.42-0.98) [n=12] | 0.85 (95% CI: 0.62-1.00) [n=12] | 1.00 (95% CI: 1.00 to 1.00) [n=12] |

Data are presented as area under the curve (AUC) value and 95% confidence interval (CI) obtained from receiver operating characteristic (ROC) curve analysis. Abbreviations: AKI, acute kidney injury; RRT, renal replacement therapy.
